# Supplementary material for: A mechanistic hydro-epidemiological model of liver fluke risk
Source: J R Soc Interface. 2018 Aug 29;15(145):20180072. doi: 10.1098/rsif.2018.0072 (PMC6127180; doi:10.1098/rsif.2018.0072)
Supplement: Supplementary Material from "A mechanistic hydro-epidemiological model of liver fluke risk" [file rsif20180072supp1.docx]

Supplementary Material

**A mechanistic hydro-epidemiological model of liver fluke risk**

*Journal of the Royal Society Interface*

Ludovica Beltrame^1*^, Toby Dunne^1^, Hannah Rose Vineer^2,3,4^, Josephine G. Walker^2,5^, Eric R. Morgan^4,6^, Peter Vickerman^5^, Catherine M. McCann^7^, Diana J.L. Williams^8^, Thorsten Wagener^1,4^

*^1^ Department of Civil Engineering, University of Bristol, Bristol, UK*

^2^ *School of Biological Sciences, University of Bristol, Bristol, UK*

^3^ *Bristol Veterinary School, University of Bristol, Bristol, UK*

^4^ *Cabot Institute, University of Bristol, Bristol, UK*

^5^ *Bristol Medical School, University of Bristol, Bristol, UK*

^6^ *School of Biological Sciences, Queen’s University Belfast, Belfast, UK*

^7^ *Epidemiology Research Unit, Scotland’s Rural College, Inverness, UK*

^8^ *Institute of Infection and Global Health, University of Liverpool, Liverpool, UK*

^*^ [ludovica.beltrame@bristol.ac.uk](mailto:ludovica.beltrame@bristol.ac.uk)

**S1:** Hydro-Epidemiological model for Liver Fluke (HELF)

HELF was implemented in the MATLAB environment (software and programming language: Matlab). The model code is available on github (<https://github.com/ludobeltrame/helf)>. Below we provide a description of HELF that follows the protocol proposed by Grimm et al. (2006):

**1. Overview**

- **Purpose:** HELF was developed as a mechanistic model to investigate the impact of weather-water-environment processes on time-space patterns of risk of infection with liver fluke under changing conditions.
- **State variables:** The model comprises 2 levels: the parasite life-cycle and the hydrologic environment. The former describes the 4 liver fluke life-cycle stages that live on pasture: eggs, miracidia, snail infections and metacercariae. Each stage (except for miracidia, that have a lifespan comparable to the temporal resolution of the model) is represented as a pool of developing cohorts of individuals. Each cohort is characterised by two state variables: number of individuals and age, where the latter is a dimensionless quantity that depends upon stage-specific development rates. With regards to the water environment, the state variable in the model is soil moisture, expressed as a saturation deficit [mm].
- **Scales**: HELF is a dynamic (daily temporal resolution) and spatially-explicit grid-based model, in which space is represented in the form of discrete grid cells. Specifically, one cell represents a 25m x 25m area. The extent of the whole spatial domain is given by the area (i.e. number of grid cells) of the catchment under consideration.
- **Process overview and scheduling**:
- First, spatially-distributed topographic information is derived from Digital Elevation Model (DEM) data in the form of a Topographic Index (TI), calculated for each grid cell comprising the given catchment.
- This is followed by discretisation of the distribution of TI values into classes, so that following computations are performed for each class instead of for each grid cell.
- The model proceeds in discrete (daily) time steps.
- At every step, first, soil moisture in each TI class is calculated as a function of the TI value of the class and the catchment average saturation deficit, which is derived based on a number of processes:
- Interception of rainfall by vegetation cover
- Infiltration of water into the upper part of the soil (i.e. the root zone)
- Actual evapotranspiration from the root zone, based on potential evapotranspiration, maximum capacity of the zone and its actual water content
- Percolation of water from the root zone to the lower part of the soil (i.e. the groundwater)
- Generation of subsurface flow and saturation-excess overland flow
- Then, soil moisture for each TI class becomes an input to the parasite life-cycle model component of HELF, where it is used, together with temperature, to calculate the relevant stage-specific development and survival rates.
- The age of cohorts in each stage is updated, based on the stage-specific development rates, to derive the number of individuals that progress to the next life-cycle stage (“matured”).
- The number of individuals in each stage is then derived as a function of: the number from the previous time step, the number of individuals that die, the number of matured, and the number of those that have developed from the previous stage.
- Finally, the number of individuals for each stage can be mapped back from TI classes to grid cells.

**2. Design concepts**

HELF is not individual-based, therefore the “Design Concepts” block as defined by Grimm et al. (2006) does not apply. However, 2 key concepts underlying the development of our model are:

- Soil moisture dynamics is simulated within HELF using TOPMODEL. This model was built with the specific characteristics of UK hydrology in mind i.e. humid-temperate catchments, where the dominant mechanism of runoff generation is surface saturation and where surface saturation is strongly related to landscape topography [Beven and Kirkby, 1979]. TOPMODEL is therefore a sensible choice for a hydrological model in the context of HELF, as long as this assumption is valid.
- In the fluke component of HELF, each life-cycle stage is represented as a pool of developing cohorts of individuals to better capture maturation progress within each stage. The underlying idea is that different cohorts are exposed to different environmental conditions and, therefore, will develop at different times.

**3. Details**

- **Initialization:** All state variables are arbitrarily initialised to zero. However, the initial saturation deficit in the root zone is a model parameter and, as such, is calibrated, as the other parameters within the hydrological component of HELF, using streamflow observations. Moreover, the initial number of embryonic eggs on each TI class is defined (as for the rest of the simulation period) by the egg scenario considered. The model is run for one year before all analyses are started, in order to limit initialisation effects (i.e. we use 1 year of warm-up).
- **Inputs:** DEM data for the catchment under study; catchment average rainfall time series; catchment average minimum and maximum temperature time series; egg scenario (i.e. one time series per TI class, defined based on local farm management factors).
- **Sub-models:**

Hydrological model component:

- TOPMODEL concepts and equations are explained in detail in e.g. Beven et al., 1995.
- Topographic Index values are calculated using the Multiple Flow Direction algorithm based on Quinn et al., 1991.
- Potential evapotranspiration is calculated using Hargreaves equation based on Allen et al., 1998 and Droogers and Allen, 2002.
- A gamma distribution is used to model the time delay in discharge generation at the catchment outlet, due to water moving through the river network (as e.g. used in Clark et al., 2008).
- Parameters and their initial ranges can be found in Table 1.

Fluke model component:

- - Given the purpose of HELF, the parasite life-cycle is driven in the model by temperature and soil moisture, which are known as the main environmental drivers of infection risk.
    - Eggs (E) develop on pasture at a temperature-dependent rate, and hatch into Miracidia (Mi) when both temperature and soil moisture conditions are suitable.
    - Progression from miracidia to the next life-cycle stage depends upon the probability of miracidia finding a snail host. This is assumed to be a function of soil moisture and temperature, as *Galba truncatula* snails are only found in poorly drained areas and are known to hibernate with cold weather and aestivate during hot dry periods. The number of snails is not explicitly modelled. Instead, increased environmental suitability is assumed to instantaneously increase snail availability, which in turn increases the probability of miracidia finding and infecting snails (we assume infection success rate of 1).
    - Snail infections (SI) develop also as a function of both soil moisture and temperature, as it is known that development within the snail may be halted due to hibernation and aestivation.
    - Despite, within snails, parasites are known to pass through several developmental stages, we do not explicitly model them in HELF and simplify the process by only representing one “snail infection” stage.
    - When snail infections emerge from snails (in the form of Cercariae), they instantaneously encyst on grass forming Metacercariae (Me).
    - Metacercariae survive on pasture and retain infectivity as a function of temperature.
  - Functions to calculate development rates for all stages and survival rates for metacercariae are derived using data and information in the literature by piecewise linear regression (Table 2 and Figure 3). For stages with both temperature and soil moisture requirements, we allow for development to progress as a function of both. The mortality rate for miracidia is set to one minus the probability of finding a snail, as miracidia either find a snail or die within 24 hours. The mortality rates for eggs and snail infections are currently assumed to be constant, as no information could be found on their dependence on environmental conditions.
  - A Weibull function is used to simulate the distribution of development times, as we assume even individuals from the same cohort, which are exposed to same environmental conditions, will not all develop at the same time [Andrews, 1999].
  - Parameters and their initial ranges can be found in Table 2, together with references.

**S2:** Epidemiological data

- VIDA dataset: yearly VIDA reports, containing monthly number of diagnoses of fasciolosis from the 15 APHA UK laboratories, are freely available from [www.gov.uk/apha](http://www.gov.uk/apha). Extracts of diagnoses associated with specific post code districts of interest are also freely available upon request.
- FEC-based dataset: FECs are calculated as number of eggs found per gram of faeces. Forty cattle were sampled and four 10 x 10g composite counts were performed per farm. Farms were subsequently classified either as positive, if at least one of the counts was positive, or negative otherwise. Based on this, 41.9% of the farms sampled were positive (this is the overall observed percentage of infection which we use to calibrate the epidemiological component of HELF for the Severn Catchment - see end of section 4.2.1.).

**S3:** Model performance metrics

The coefficient of determination R^2^ is employed as a standard measure to evaluate how the hydrological component of HELF reproduces the dynamics of observed streamflow with an emphasis on the peaks.

It is calculated as follows: $R^{2}= \frac{{(\sum_{i=1}^{N} ({obs}_{i}-\overline{obs})({sim}_{i}-\overline{sim}))}^{2}}{\sum_{i=1}^{N} {({obs}_{i}-\overline{obs})}^{2} \sum_{i=1}^{N} {({sim}_{i}-\overline{sim})}^{2}}$

For evaluating the epidemiological component instead, as the variable we simulate is different from the available observations, we are more generally interested in the agreement between the two. Therefore, we use Pearson correlation coefficient.

**S4**: Cross-validation for the epidemiological component of HELF

- VIDA data: we randomly divide the time series into 5 sub-sets and repeat calibration and validation 5 times, using every time (5-1) sub-sets for calibration and the remaining one for validation.
- FEC data: given the limited number of data points, we perform a leave-one-out cross-validation here, consisting of sequentially removing one data point only, refitting the model to the rest of the data, and predicting the value of the ignored observation. The residuals resulting from this process are used to assess the predictive ability of the model based on the mean absolute cross-validation error.

**Figure S1**: Risk of infection seems overestimated in sub-areas A2 and A5. A first potential reason for this mismatch is that these two sub-areas were significantly drier compared to the others in 2014, but this is currently not accounted for in our model. In fact, we are currently neglecting the spatial variability of rainfall over the catchment, by driving HELF with one rainfall time series only (average of the time series from the grid cells overlapping with the catchment area).


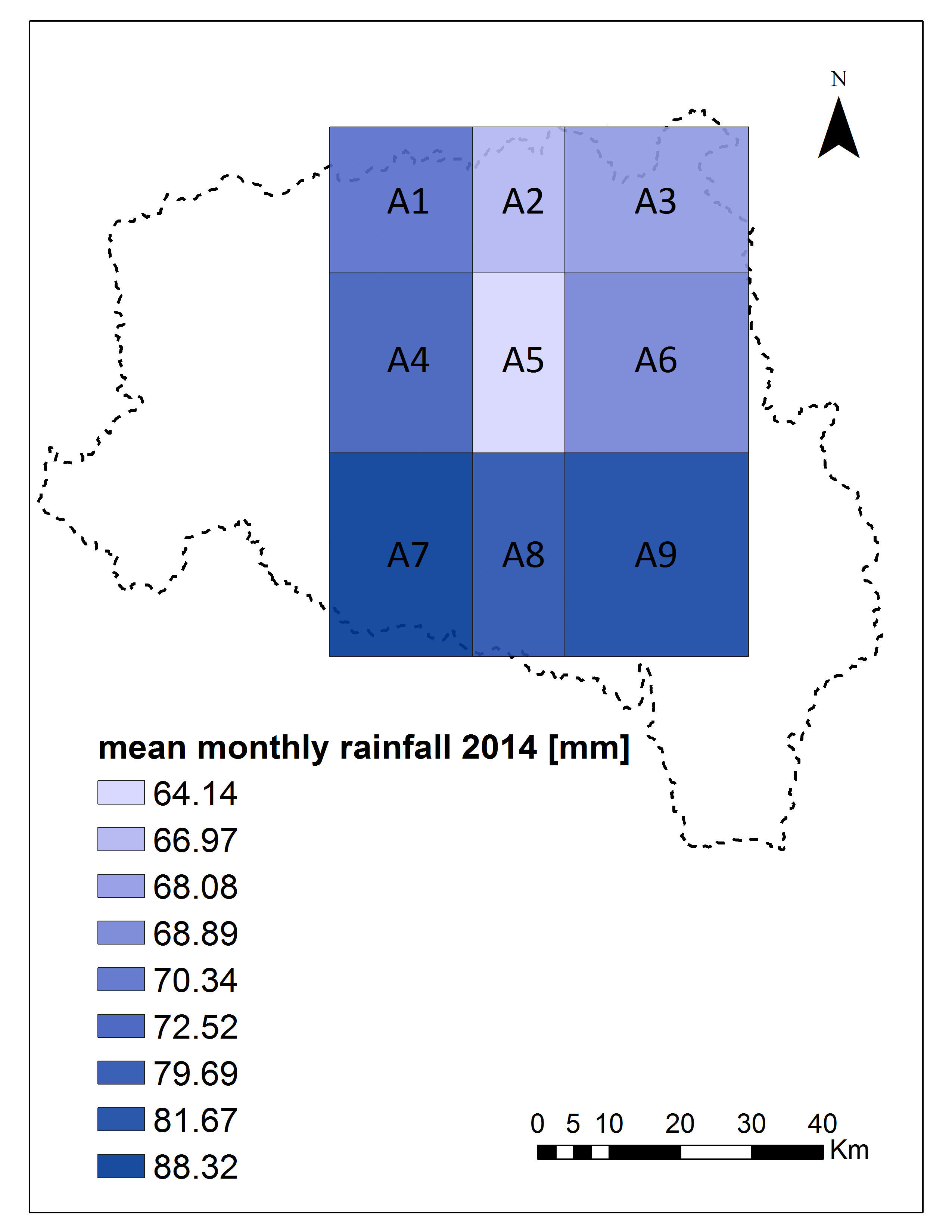


**Figure S2**: Risk of infection seems overestimated in sub-areas A2 and A5. A second potential reason for this mismatch is related to how suitable these areas are in terms of soil pH for development progress of the parasite life-cycle. In fact, *Galba truncatula* snails (main intermediate host for *F. hepatica*) are known to prefer slightly acidic soils, i.e. soil pH between 5.5 and 6.5 [Ollerenshaw, 1971]. Only 25.5% of the area of A2 has slightly acidic soil [NSRI LandIS]. The figure is higher for A5 (40.4%), but still lower than the average across the 9 sub-areas (43.7%). However, risk of infection in HELF is currently calculated based on temperature and soil moisture only, neglecting potential effects of soil pH on snail presence.


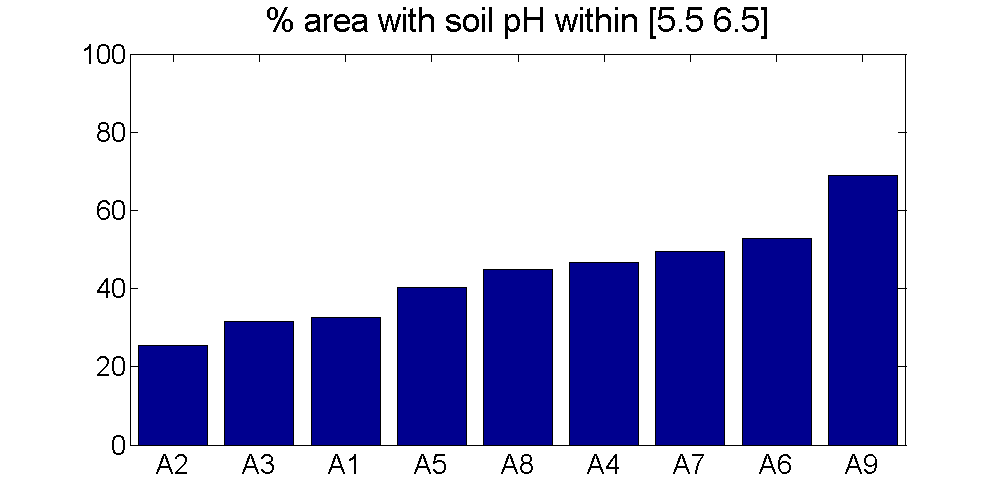


Finally, we are currently driving HELF with a scenario which assumes continuous livestock grazing and absence of disease management over the catchment. This could also result in mismatches depending on the real farm management strategies in use, e.g. housing of cattle or administration of treatment may result in lower observed risk than what simulated using our model.

**Figure S3**: Temporal comparison of the risk pattern obtained using the Ollerenshaw Index with pasture contamination simulated with HELF, over the whole simulation period (2000-2010) for the Tawe Catchment.


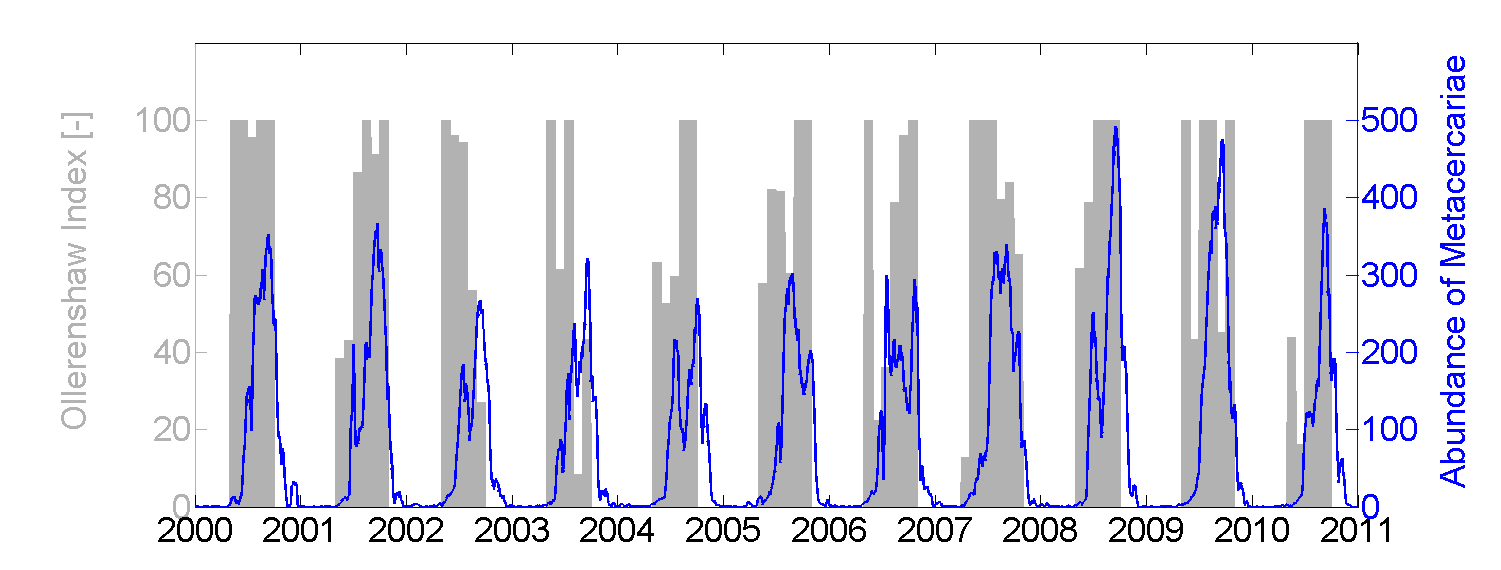


**References**

- Allen et al. (1998), Crop evapotranspiration - Guidelines for computing crop water requirements, FAO irrigation and drainage paper 56.
- Andrews (1999), The life cycle of Fasciola hepatica. In: Dalton, JP (Ed.), Fasciolosis. CABI Publishing. pp: 1-29.
- Beven and Kirkby (1979), A physically based, variable contributing area model of basin hydrology, *Hydrological Sciences Bulletin* 24:43-69. http://dx.doi.org/10.1080/02626667909491834.
- Beven et al. (1995), TOPMODEL, in Computer Models of Watershed Hydrology, edited by VP. Singh, pp. 627-668, Water Resour. Publ., Colorado.
- Clark et al. (2008), Framework for Understanding Structural Errors (FUSE): A modular framework to diagnose differences between hydrological models, *WRR* 44:W00B02. http://dx.doi.org/10.1029/2007WR006735.
- Droogers and Allen (2002), Estimating reference evapotranspiration under inaccurate data conditions. *Irrigations and Drainage Systems* 16:33-45.
- Grimm et al. (2006), A standard protocol for describing individual-based and agent-based models, *Ecological Modelling* 198:115-126. http://dx.doi.org/10.1016/j.ecolmodel.2006.04.023.
- National Soil Resources Institute Land Information System (NSRI LandIS), Cranfield University, UK. <http://www.landis.org.uk>.
- Ollerenshaw (1971), Some observations on the epidemiology of fascioliasis in relation to the timing of molluscicide applications in the control of the disease, *Veterinary Record* 88:152-64. http://dx.doi.org/10.1136/vr.88.6.152.
- Quinn et al. (1991), The prediction of hillslope flow paths for distributed hydrological modelling using digital terrain models, *Hydrological Processes* 5:59-*79*. <http://dx.doi.org/10.1002/hyp.3360050106>.
